# Current Perspectives on the Use of Meditation to Reduce Blood Pressure

**DOI:** 10.1155/2012/578397

**Published:** 2012-03-05

**Authors:** Carly M. Goldstein, Richard Josephson, Susan Xie, Joel W. Hughes

**Affiliations:** ^1^Kent State University, Kent, OH 44242, USA; ^2^Summa Health System, Akron, OH 44309, USA; ^3^Harrington Heart & Vascular Institute, University Hospitals Case Medical Center and Department of Medicine, Case Western Reserve University School of Medicine, Cleveland, OH 44106, USA; ^4^Rice University, Houston, TX 77005, USA

## Abstract

Meditation techniques are increasingly popular practices that may be useful in preventing or reducing elevated blood pressure. We reviewed landmark studies and recent literature concerning the use of meditation for reducing blood pressure in pre-hypertensive and hypertensive individuals. We sought to highlight underlying assumptions, identify strengths and weaknesses of the research, and suggest avenues for further research, reporting of results, and dissemination of findings. Meditation techniques appear to produce small yet meaningful reductions in blood pressure either as monotherapy or in conjunction with traditional pharmacotherapy. Transcendental meditation and mindfulness-based stress reduction may produce clinically significant reductions in systolic and diastolic blood pressure. More randomized clinical trials are necessary before strong recommendations regarding the use of meditation for high BP can be made.

## 1. Introduction: Meditation Techniques as Treatments for Elevated Blood Pressure

According to worldwide estimates, hypertension affects approximately 1 billion people, resulting in 7.1 million attributed deaths per year [1]. In the United States, nearly half of all adults have blood pressure (BP), expressed in terms of systolic (SBP) over diastolic blood pressure (DBP), in the prehypertensive (SBP of 120–139 or DBP of 80–89) or hypertensive (SBP > 140 or DBP > 90) range [2, 3]. As one of the most widespread, least controlled diseases around the world, hypertension poses a threat to adults from all cultures and lifestyles. Factors such as improved treatment, pharmacologic interventions, preventative measures, and lifestyle changes have contributed to a 60% decrease in age-adjusted death rates from stroke and a 50% decrease in age-adjusted death rates from coronary heart disease in the United States since 1972. However, despite these improvements, BP control among American adults still remains suboptimal. For example, two-thirds of hypertensive individuals are not being controlled to recommended BP levels. Furthermore, approximately 30% of American adults are unaware of their hypertension, and over 40% of those with hypertension are not receiving treatment [3].

Current treatment guidelines for hypertension include antihypertensive medications and health-promoting lifestyle modifications such as weight reduction, the DASH eating plan (increased fruits and vegetables, and low fat dairy products with reduced saturated and total fat), reduced dietary sodium, increased physical activity, and moderation of alcohol consumption. Ideally, antihypertensive medications and lifestyle modifications successfully reduce BP to optimal levels. However, despite the effectiveness of antihypertensive medication [4], adherence to medication regimens is often poor and interferes with the goal of reducing BP [5, 6]. In addition, hypertensive medications can produce troublesome side effects such as insomnia, sedation, dry mouth, drowsiness, impotence, and headaches [4]. Due to difficulty adhering, side effects, and prescription drug costs, hypertensive individuals may desire a nonpharmacologic intervention to avoid or complement their antihypertensive medication regimen. Therefore, whereas continued improvement in pharmacologic treatments is necessary, these advancements must be complemented by nonpharmacological approaches to BP control. Toward that end, mind-body interventions such as relaxation, stress management, and meditation—whether used alone or in combination with lifestyle modifications—have been evaluated as potential treatments for high BP (refer to Table 1 for an overview of the major types of mind-body interventions). Ample evidence exists regarding the effects of relaxation and stress management on BP to draw some conclusions, which are discussed below. However, less is known regarding the potential of meditation as an intervention for hypertension. The purpose of this focused paper is to evaluate the evidence that meditation is an effective intervention for lowering elevated BP (refer to Table 2 for a summary of studies in the literature search).

## 2. Mind-Body Interventions

Relaxation therapies for hypertension have been evaluated for over 30 years with disappointing results. For example, the Hypertension Intervention Pooling Project found no treatment effect for SBP and a small effect for DBP [22]. In a study by Irvine and Logan, relaxation therapy produced effects equal to a group that received support therapy, and the relaxation group did not produce a larger decrease in BP [23]. Positive results sometimes observed for relaxation can often be explained by methodology [24]; that is, individuals with higher baseline BPs tend to benefit more than individuals with lower baseline BPs, and repeated monitoring of BP appears sufficient to reduce BP levels [24, 25]. Overall, relaxation techniques, the most common being progressive muscle relaxation (PMR), are not considered effective methods for treating hypertension [26]. Consequently, PMR can even be used as a control group for randomized controlled trials of other mind-body interventions.

In contrast, stress-management therapies have had some success reducing BP [27–30]. In a meta-analysis, multicomponent stress management treatments were more effective in reducing BP (13.5 mm Hg SBP and 3.4 mm Hg DBP) than sham treatments, whereas single-component therapies (e.g., relaxation alone) did not produce significant results [31]. Another meta-analysis reported that multicomponent stress-management therapies were more effective in reducing BP than single-component relaxation-based therapies [32]. The Canadian Coalition for High Blood Pressure Prevention and Control has recommended multicomponent stress management be considered for hypertensive individuals whose stress appears to contribute to their hypertension [30].

Despite the promise of multicomponent stress-management interventions, implementation has not been widespread. Historically, the field of behavioral medicine has taken a keen interest in the contribution of stress to elevations in BP. Naturally, evidence that stress contributes to elevated BP and hypertension was followed by attempts to treat stress in order to reduce BP. However, stress-management interventions for high BP are neither widely available nor commonly practiced. Although we cannot be certain, we speculate the association of stress management programs with mental health treatment may introduce stigma and reduce patient acceptance of stress-management approaches. The expense of treatment programs and relative scarcity of healthcare professionals qualified to provide stress management to patients with high BP may also contribute to the limited implementation of multicomponent stress management programs as a uniquely behavioral medicine solution to hypertension.

Another mind-body intervention for high BP is meditation. It appears to be a promising option, as meditation is portable and can be practiced independently of structured treatment programs, although evaluation in clinical trials obviously requires a clear treatment protocol. Meditation has less association with mental health stigma and may be a more acceptable intervention than stress-management treatments in many cultures.

The most widespread types of meditation interventions can be roughly grouped into mantra meditation, such as transcendental meditation (TM) and mindfulness meditation. Mindfulness meditation involves an attitude of openness, acceptance, and reflection rather than impulse and judgment toward the practitioner's current experiences, as well as the observation of thoughts, feelings, and the external world alike through calm, detached sensory awareness [33]. Mantra meditations focus on a word, phrase, or concept. The mantras often have soft sounds, like “Om,” and these words are thought to produce different vibrations in different people, producing various effects on the individual [34]. In the TM technique, the mantras, which are used for sound value rather than meaning, become increasingly secondary in experience and eventually disappear, while self-awareness becomes primary in experience as the practitioner transcends to a state of pure consciousness [35].

Mindfulness—described as a calm awareness of one's body, mind, and environment that embodies an interaction between nonjudgmental acceptance and focuses on the present moment—has existed for over 2,500 years and can be found in numerous religions, cultures, meditation techniques, and psychotherapies [36]. Although it is the 7th step of the Noble Eightfold Path in Buddhism, there is nothing inherently religious about mindfulness as it is mostly taught completely independent from any religious doctrine. Mindfulness meditation research became formalized in 1979 when Dr. Jon Kabat-Zinn founded the MBSR program at University of Massachusetts-Amherst to treat the chronically ill [37]. Other practices and interventions that utilize the concept of mindfulness include yoga [38], acceptance and commitment therapy [39], dialectical behavior therapy [40], Tai Chi [41], and Qigong [42].

### 2.1. Transcendental Meditation

TM has been extensively studied as a meditation therapy for high BP. In one study, the feasibility and efficacy of TM and progressive muscle relaxation (PMR) were tested via a subgroup analysis by sex and level of hypertension risk in older African Americans [17]. Compared to control subjects who underwent lifestyle modification education only, TM produced significant declines in BP after 3 months for both men (by 12.7 mm Hg SBP and 8.1 mm Hg DBP) and women (by 10.4 mm Hg SBP and 5.9 mm Hg DBP). In contrast, women practicing PMR failed to show significant declines, while men practicing PMR experienced significant declines solely in DBP (by 6.2 mm Hg) [17]. An earlier randomized controlled trial of TM by the same authors reported that 20 elderly patients who were treated with TM exhibited a 12.4 mm Hg drop in SBP, compared to a 2.4 mm Hg reduction for patients in the control group [21].

The short-term efficacy of TM and PMR in treating mild hypertension was also evaluated in 127 African American men and women aged 55 to 85 years, compared with a lifestyle modification education control program [18]. TM reduced SBP (10.7 mm Hg) and DBP (4.7 mm Hg), which was significantly greater than those observed for relaxation (4.7 mm Hg SBP and 3.3 mm Hg DBP). Between the two stress-reducing approaches, TM was about twice as effective as PMR. Later, Schneider and colleagues [19] conducted another study following African American hypertensive individuals over one year while they underwent TM, PMR, or conventional health education classes as a control. The TM group experienced greater decreases in SBP and DBP than the PMR or control groups, as well as reduced use of antihypertensive medication, relative to increases for PMR and the control group. Consequently, the TM program may be particularly useful in the long-term treatment of hypertension in African Americans, for whom many of these effects have been demonstrated. Schneider and colleagues also conducted a recent meta-analysis, which revealed that, compared with combined controls, the TM group showed substantial decreases in all-cause mortality, cardiovascular mortality, and cancer-related mortality [20].

In another study [16], unmedicated patients with hypertension underwent TM-based training (treatment group), TM-based training without a mantra (placebo control group), or no training (no-treatment control group). Compared with the no-treatment controls, modest BP declines were observed in both the treatment and placebo control groups, with DBP percentage showing a significant decrease [16]. The similarity in effectiveness of TM training and TM training without a mantra could be attributed to the fact that both were in effect “meditation" groups or that changes in BP were due to another factor. A meta-analysis that only included high-quality assessments—as determined by 11 factors, which included participant selection, randomization, blinding, full description of the therapeutic intervention, and appropriate measurements of BP—found TM, compared to controls, associated with clinically meaningful reductions of 4.7 and 3.2 mm Hg in SBP and DBP, respectively [43]. TM has also appeared to reduce carotid arteriosclerosis in African Americans [12] and a 4.8 mm Hg drop in ambulatory DBP among white males treated with TM [13].

A study assessing the effects of TM on BP, psychological distress, and coping among university students was also the first randomized clinical trial to demonstrate that TM significantly increased coping and reduced BP in association with lessened psychological distress in a hypertension risk subgroup. The TM program may decrease the risk for developing hypertension in young adults [14]. TM also reduced resting BP among adolescent African Americans with high normal BP (with a resting SBP ≥85th and ≤95th percentile) over two months, with larger declines than those in a health education control group, demonstrated during both at rest and during acute laboratory stressors [11]. In another study on African American adolescents with high normal systolic BP, the 4-month TM group showed greater declines in daytime SBP (*P* < 0.04) and DBP (*P* < 0.06) compared to the health education control group, further exhibiting a beneficial impact of TM in youth at risk for hypertension. This study is of particular interest due to its utilization of ambulatory 24-hour BP monitoring, which not only tends to be relatively free of placebo effects and to be highly reproducible but also records BP regularly over a prolonged time period in the participants' natural environments, thus increasing sensitivity to changes in average BP and providing a more reliable measure of overall BP [15]. Ambulatory studies like the one produced here by Barnes and colleagues are valuable because they generate the potential to measure treatment effects out of the laboratory and in day-to-day life, which may allow generalization of treatment effects.

### 2.2. MBSR

Mindfulness-based stress reduction (MBSR), a subset of mindfulness meditation that has been standardized and manualized, is said to treat depression and anxiety, lower stress, and treat health conditions like hypertension. MBSR is a program that utilizes meditation and stress management techniques. Originally founded by Dr. Jon Kabat-Zinn, The Center for Mindfulness in Medicine, Health Care, and Society at the University of Massachusetts Medical School (http://www.umassmed.edu/cfm/) has treated over 19,000 patients with MBSR.

MBSR was originally developed and used in a behavioral medicine setting for individuals with chronic pain [44] and typically consists of eight 2.5-hour weekly group sessions. These sessions contain instruction and practice in mindfulness meditation, as well as conversations of stress, coping, and homework assignments. Students learn a range of mindfulness skills including body scan exercises, sitting meditation, and yoga exercises. Homework consists of practicing these skills for at least 45 minutes per day, 6 days per week, in addition to practicing mindfulness skills during group meetings. The program concludes with an 8-hour intensive mindfulness retreat with a therapist. During and after the program, students are encouraged to pay mindful, nonjudgmental attention to daily activities like walking, eating, and talking. One goal is for participants to see that most sensations, emotions, and thoughts are short-lived and do not require immediate suppression.

Recent studies have evaluated the effectiveness of MBSR for reducing BP, as well as breathing awareness meditation (BAM), a primary exercise in MBSR, in producing declines in BP among differing populations. In one study, 121 African American ninth graders (with a resting SBP >50th percentile and <95th percentile) underwent BAM, life skills training, or health education [8]. Among the three behavioral intervention approaches, only BAM produced significant decreases in 24-hour SBP. Another study also conducted among 166 African American ninth graders at increased risk for essential hypertension compared the treatment effects of BAM, the Botvin LifeSkills Training, or a health education control [9]. Significant group differences emerged, with the BAM group exhibiting the greatest decreases in SBP, DBP, and heart rate.

MBSR has also shown some potential for lowering BP in individuals with elevated BP. A recent study comparing the effects of MBSR versus PMR on prehypertensive adults found that MBSR produced significant reductions in SBP and DBP. A 4.9 mm Hg reduction in clinic SBP was observed in the MBSR group compared to 0.7 mm Hg in the PMR group, and MBSR produced a 1.9 mm Hg reduction in DBP compared to a 1.2 mm Hg increase for PMR [10]. The results were qualitatively similar to reductions in BP reported in a meta-analysis of TM [45], as well as the reduction observed in the PREMIER trial of comprehensive lifestyle modification for high BP [46]. In another study, adult female posttreatment cancer patients who underwent MBSR did not experience significant differences in BP compared to the waitlist control group [7]. However, when patients were analyzed by “higher BP” and “lower BP” groups through a median split based on BP readings during the first week of treatment, “higher BP” MBSR participants had lower SBP compared to controls at the end of the MBSR program. Given the normotensive sample and preliminary results due to methodological limitations of the study (e.g., results may have been an effect of regression to the mean), more well-designed trials are needed to evaluate the utility of MBSR in reducing clinically elevated BP [7].

## 3. Methodological Considerations

A report on meditation techniques conducted by the United States Agency for Healthcare Research and Quality (AHRQ) evaluated the methodological quality of 286 randomized controlled trials employing meditation practices in a variety of populations [33]. Studies were evaluated using the Jadad scores, as the Jadad scale is the most commonly used assessment scale of methodological quality of randomized controlled trials in health care research [47]. Scores (on a scale of 1–5, from lowest to highest methodological quality) are based on reported method of randomization, double-blinding, and description of withdrawals and dropouts, with low scores indicating a higher risk of bias [48]. The quality of meditation trials was evaluated to be poor overall, with only 14% being rated high quality (i.e., Jadad scores ≥3 points); the studies reviewed were found to have too many qualitative or observational reports, limited descriptions of participant characteristics (including if the inclusion criteria required an official diagnosis of prehypertension or hypertension), small sample sizes, inadequately described blinding procedures and randomization, lack of control groups, insufficient followup periods, limited reporting of intention-to-treat statistical analyses, and inadequately described losses to followup [33]. Furthermore, much of the research published on specific forms of meditation has been conducted by the organizations that create or disseminate those specific forms of meditation. Although the methodological quality of this research is improving, meditation techniques should be tested by independent research teams who have no association with organizations promoting a particular approach to meditation. Recent additions to the literature have increasingly adhered to the CONSORT recommendations (Consolidated Standards of Reporting Trials; 49). There are many methodological improvements that can be made. For example, conflict of interest and researcher bias can be minimized by collaborative efforts with outside institutions having independent oversight of data collection and analysis, independent replication, rigorous blinding, allocation concealment, randomization, and selection of a suitable control condition.

## 4. Future Directions and Studies

Future research targeting meditation interventions must begin by adequately defining the role of mindfulness or other concepts and components in meditation and delineating intentions for applications to the study population. Meditation treatments should be standardized and manualized as much as possible to ensure maximal external validity. Additionally, similarities and differences between kinds of meditation interventions should be highlighted within publications. The role of mindfulness and meditation in a given intervention should be explained. Furthermore, assessments that target measurements of the construct, mindfulness, and the process through which mindfulness is achieved, meditation, should be refined and psychometrically validated in medical populations and healthy controls.

Future studies should clearly outline their inclusion and exclusion criteria, with efforts to extend meditation interventions to hypertensive individuals who otherwise belong to understudied populations. Including a variety of populations makes the examination of potential moderators such as ethnicity possible. Aims should include using larger sample sizes and continuing with the disease-specific approach to interventions, implying the use of strict inclusion criteria requiring participants must be diagnosed with either prehypertension or hypertension to participate. There may need to be a better selection of control protocols, with preference given to similar interventions that have been validated to not produce the results experimenters expect to find (or not) in the experimental condition. In addition, studies defining dose response would be substantially beneficial in helping not only to confirm the correlation-effect link, but also to determine how much of an intervention produces both statistically and clinically significant effects. This would facilitate wider dissemination and scalability.

Procedural and statistical methodology must be explicitly outlined so the studies can be critiqued and replicated and so articles published from the studies can be included in reviews and meta-analyses. With improved adoption of the CONSORT guidelines [49], better systematic comparisons of effects of different mindfulness interventions can be established. Hopefully, as the methodology and reporting of these studies is strengthened and clarified, scientists will be able to adopt more experimental designs, ultimately optimizing the ability to make causal inferences.

Meditation is an intervention for hypertension and prehypertension that is perhaps best characterized as being in its adolescence. There is clearly considerable promise, with a variety of studies demonstrating efficacy in the short-term reduction of BP similar to that achieved with single-agent drug therapy. On the other hand, many of these studies are potentially biased due to lack of blinding, inadequate baseline measurements of BP, and limited followup. All meditation techniques are not created equal, and few studies have directly compared one technique to another. More importantly, there has been essentially no evaluation to determine what may be the essential components of a putatively successful methodology or if an entire “standard” approach is required. This has major implications for scalability, particularly in resource-limited settings where both clinical staff time and patient meditation environment and time may be constrained. Hypothesis-driven mechanistic studies are rare and, if well conceived and executed, could dramatically advance the field. Potential mechanisms of action include alterations in the autonomic nervous system, with changes in the sympathovagal balance favoring the latter. Perhaps meditation affects mood in hypertensives; in other settings depression has been associated with physical inactivity and altered eating patterns, both of which may affect BP.

Hypertension is paradigmatic of a chronic disease, with clinical sequelae typically developing after years of elevated BP. Long-term followup, after the acute intervention is complete but, while the patient is still employing meditative techniques, is essential. Perhaps “booster doses” of instruction will be required. While prima facie meditation would appear to be free of side effects, few studies have systematically evaluated their presence and consequences. It is possible that the most significant side effect may be procedural, where meditation is simplistically viewed only as an alternative or substitute for antihypertensive drugs. Pharmacotherapy of hypertension frequently involves multiple drugs, particularly in those with substantially elevated baseline pressure. It is unlikely that meditation will be effective as monotherapy in all (and perhaps most of) patients with established hypertension. A therapeutic approach of multimodality treatment, wherein meditation is truly viewed as complementary to drug treatment, is an important underexplored area, with the potential to expand the number of individuals who could both benefit from meditative techniques and achieve improved BP control.

Perhaps the greatest potential benefits of meditation techniques in the treatment of individuals with hypertension are in developing countries. Many of these countries are experiencing large population growth and with the increasing penetration of a Western lifestyle come both increased caloric and sodium intake and decreased physical activity. In these circumstances, cardiovascular diseases, particularly hypertension, are assuming increased prevalence. Meditation techniques, if they can be delivered efficiently and effectively, may prove to be valuable tools to treat the growing epidemic of hypertension, particularly if they eliminate the inconveniences of laboratory monitoring or prescription refills and indeed have few and rare side effects.

It is our hope that this overview of meditation techniques has highlighted prior successes, outlined the limitations existent in the field today and provided inspiration and guidance to move the field forward with mechanistic, specifically detailed, and long-term studies in the future.

Considering the current healthcare system in the United States, it is possible for mindfulness interventions to be implemented as both prevention and treatment programs (pending confirmation of their effectiveness). Most mindfulness interventions can be taught in a group format, which reduces the cost on participants and the burden on clinicians. As more treatments are standardized and their efficacy can be demonstrated in clinical trials, insurance companies may be more inclined to fund mindfulness training. Longitudinal studies must also be executed to determine if mindfulness can act as a protective factor against an array of psychosocial and medical ailments. Positive results may indicate that mindfulness interventions could produce a clinically significant resiliency or protection against problems requiring care from mental health and medical professionals. As a promising construct in complementary and alternative medicines, there is a strong possibility that mindfulness could become a component of effective interventions designed to prevent hypertension and lower suboptimal BP.

## Figures and Tables

**Table 1:** Major types of mind-body interventions.

Major types of mind-body interventions	Subtypes	Description	Setting	Certification to administer	Dose	Standardization
Relaxation therapy	Progressive muscle relaxation (PMR), biofeedback, relaxation-assisted biofeedback, autogenic training	Uses relaxation techniques to achieve physical and mental relaxation, often coupled with breathing exercises or mental imagery	Group or individual	Varies (health professionals, psychologists, therapists, etc.)	Varies (e.g., weekly sessions with homework assignments)	Not standardized
Stress-management therapy	N/A	Adjusts behavioral and psychological responses to stress through cognitive behavioral interventions	Group or individual	Varies	Varies	Not standardized
Zen meditation	N/A	Focuses attention on counting deep breaths or koans (riddles irresolvable by logic) to cultivate awareness	Mostly individual	Zen practitioner	Varies	Not standardized
Transcendental meditation (TM)	N/A	Takes attention beyond normal thinking processes until thought is transcended and a state of pure consciousness is achieved, beginning with repetition of a mantra	Mostly individual	Certified instructor through the Maharishi Vedic Education Foundation	Technique learned in a 7-step course or through personal instruction, practiced 15–20 min twice/day	Standardized
Mindfulness-based stress reduction (MBSR)	N/A	Uses meditation and stress-management techniques, including mindfulness skills, such as coping, sitting meditation, and yoga, to improve physical and emotional well-being	Group sessions with individual practice	Varies	8 weekly 2.5 hr. sessions, with at least 45 min. of daily practice 6 days/week, concluding with an 8 hr. mindfulness retreat with a therapist	Standardized

**Table 2:** Literature search overview.

Meditation study overview	Sample size, population	Intervention/dose	Control	Length of baseline, no. of BP readings	Randomized, blinded	Therapists' training	Results	F/U
Mindfulness [7]	70 (normotensive, female posttreatment cancer patients, age ≥18)	8 wk. MBSR	Passive (waitlist)	3 readings taken at 3-min. intervals	Not randomized, NR if blinded	Clinical psychologist with over 10 yrs. of experience	No significant difference in BP between MBSR group and control; when patients were analyzed by “higher BP" and “lower BP" groups based on BP readings at week 1, “higher BP" MBSR participants had lower SBP compared to controls at week 8	None
Mindfulness [8]	121 (African American ninth graders, resting SBP between 50th and 95th percentiles)	Life skills training, health education, or Breathing Awareness Meditation (BAM), with 10-min. in-school and at-home sessions every day for 3 mos.	None	4 readings taken within 10 min. (first reading discarded) over 3 consecutive days	Randomized, single-blind	Health/physical education teachers trained and certified by program instructors	Only the BAM group showed significant decreases in 24-hour SBP	3 mos.
Mindfulness [9]	166 (African American ninth graders, resting SBP between 50th and 95th percentiles)	Botvin LifeSkills Training or BAM, with 10-min. in-school and at-home sessions every day for 3 mos.	Active (health education)	3 readings taken within 10 min. (first reading discarded) over 3 consecutive days	Randomized, NR if blinded	Health education teachers trained by program instructors	BAM group showed greatest decreases in SBP, changes in overnight SBP, DBP, and heart rate (significant group differences)	None
Mindfulness [10]	56 (adults aged 30–60 yrs., 91% Caucasian, BP in the prehypertensive range, 120–139 mm Hg SBP, or 80–89 mm Hg DBP, unmedicated)	MBSR for 8 wks.	Active (PMR training)	3 readings taken at 5-min. intervals, followed by 2 additional measurements within 2 wks.	Randomized, single-blind	MBSR and PMR therapists	MBSR produced significant decreases in clinic SBP (by 4.9 mm Hg) and DBP (by 1.9 mm Hg)	None
TM [11]	35 (adolescents with high normal BP aged 15–18 yrs., 34 African Am., 1 Caucasian Am., resting SBP ≥85th and ≤95th percentile)	TM, with 15 min meditation sessions twice/day for 2 mos.	Active (health education)	3 consecutive occasions, length of baseline NR	Randomized, NR if blinded	NR	TM group showed greater decreases in resting SBP and in SBP during acute stressor	None
TM [12]	60 (African American adults, aged >20 years; with high normal BP of 130–139/80-85, stage 1 hypertension BP of 140–159/90–99, or stage II hypertension BP of 160–179/100–109)	TM for 6–9 mos. (average intervention period of 6.8 ± 1.3 mos.)	Active (CVD risk factor prevention education program)	3 readings taken at each of 3 consecutive visits (last 2 visits were averaged)	Randomized, single-blind	Certified instructors from the African American community	Both groups had significant decreases in BP (TM group by 7.77 ± 10.34 mm Hg SBP and 3.5 ± 7.6 mm Hg DBP, control group by 6.74 ± 12.8 SBP and 5.9 ± 8.6 DBP), but only the BP decrease in TM group was associated with corresponding decrease in carotid intima-media thickness)	None
TM [13]	39 (normotensive Caucasian Am. male adults, mean age of 24.6 yrs.)	TM for 4 mos.	Active (cognitive-based stress education)	BP measured every 4 min. for 20 min.	Randomized, single-blind	Qualified TM instructor	TM decreased ambulatory DBP by 4.8 ± 2.4 mm Hg (8.8 ± 3.0 mm Hg in high-compliance subgroup)	None
TM [14]	298 (university students, BP <140/90 and > 90/60 mm Hg, with 159 in a hypertension risk subgroup for having SBP>130 mm Hg, DBP>85 mm Hg, or other risk factors)	TM for 3 mos.	Passive	3 readings taken at 1-min. intervals (last 2 readings were averaged)	Randomized, single-blind	Research staff and TM instructional staff	In the hypertension risk subgroup, TM significantly reduced SBP by 5.0 mm Hg and DBP by 2.8 mm Hg; reductions in overall sample were not significant. TM produced significant improvements in total psychological distress, anxiety, depression, anger/hostility, and coping.	None
TM [15]	100 (African American adolescents aged 16.2 ± 1.3 yrs., with high normal SBP)	TM for 4 mos.	Active (health education control with lifestyle education sessions)	Readings taken from 6AM–11PM every 20 min. (daytime) and 11PM-6AM every 30 min. (nighttime) over 24 hrs.	Randomized, NR if blinded	NR	TM group showed greater declines in daytime SBP (*P* < 0.04) and DBP (*P* < 0.06) compared to the health education control group	4 mos.
TM-based [16]	41 (adults aged 22–62 yrs., with essential hypertension, unmedicated, ≥100 mm Hg arterial pressure)	SRELAX group received training over 5 wkly. sessions based on TM (including mantra), with 15–20 min meditation sessions twice/day	Both passive and active (NSRELAX placebo group had same training, no mantra)	5 readings taken at 1 min. intervals	Randomized, single-blind	Experienced TM instructor	Both SRELAX and NSRELAX modestly decreased BP, with significant decrease only in DBP	3 mos.
TM or relaxation [17]	127 (hypertensive African Am. adults aged 55–85 yrs., SBP ≤179 mm Hg, DBP 90–104 mm Hg)	Transcendental Meditation (TM) or progressive muscle relaxation (PMR), with 1 wk. initial instruction and 1.5 hr. monthly followups for 3 mos.	Active (lifestyle modification)	4 readings over 1-2 mos.	Randomized, single-blind	NR	TM significantly decreased BP in both women (SBP by 10.4 mm Hg, DBP by 5.9 mm Hg) and men (SBP by 12.7, DBP by 8.1); PMR only decreased DBP significantly in men (by 6.2)	3 mos.
TM or relaxation [18]	127 (African Am. adults aged 55–85 yrs., with mild hypertension, SBP ≤189 mm Hg, DBP 90–109 mm Hg, final baseline BP ≤179/104 mm Hg)	TM or PMR, with 1 wk. initial instruction and 1.5-hr. monthly followups for 3 mos.	Active (lifestyle modification)	3 readings taken at one visit	Randomized, single-blind	Africa Am. instructors qualified to teach either TM or PMR	TM decreased SBP by 10.7 mm Hg, DBP by 6.4 mm Hg (both significantly greater decreases than those in PMR)	3 mos.
TM or relaxation [19, 20]	150 (African Am. adults, mean age of 49 ± 10 yrs., SBP 140 to 179 mm Hg, DBP 90–109 mm Hg)	TM or PMR	Active (conventional health education)	3 readings taken within 1 hr. at each of 5 sessions over 1 mo.	Randomized, single-blind	NR	TM decreased SBP by 3.1 mm Hg, DBP by 5.7 mm Hg (greatest decrease of all groups); TM decreased use of antihypertensive medication (relative to increases in other groups)	1 yr.
TM, mindfulness, or relaxation [21]	72 (elderly retirement-age adults, mean age of 81 yrs.)	TM, mindfulness training (MF), or mental relaxation	Passive	3 readings taken at 2-min. intervals (only SBP reported)	Randomized, single-blind	21 trained instructors (professionals, graduate students, and college seniors)	TM decreased SBP by 12.4 mm Hg (greatest decrease of all groups), and survival rate was 100% (compared to the second highest, 87.5% in MF) after 3 yrs.	3 yrs.
